# A preliminary study of stroke patients and attention: Exploring the role of spirituality and religiousness on cognition

**DOI:** 10.1192/j.eurpsy.2021.1138

**Published:** 2021-08-13

**Authors:** V. Giannouli, K. Giannoulis

**Affiliations:** 1 Institute Of Neurobiology, Bulgarian Academy of Sciences, Sofia, Bulgaria; 2 Faculty Of Theology, Aristotle University of Thessaloniki, Thessaloniki, Greece

**Keywords:** Attention, stroke, religiousness, Spirituality

## Abstract

**Introduction:**

Spirituality and religiousness are not so far extensively investigated in patients after stroke.

**Objectives:**

The aim of this preliminary study is to explore whether self-reports in two questionnaires measuring the personal experience of spirituality and religiousness can influence cognition and more specifically performance on neuropsychological tests examining attention.

**Methods:**

Fifteen male stroke patients participated voluntarily one year after their hospitalization. The mean age of the patients was 75.58 years (SD = 7.50, range 61-90), level of education 15.47 years (SD = 3.82). In addition to that, fifteen controls with similar demographics, free of physical and mental diseases, were also examined. Depressive symptoms of the participants were assessed with the 15-item Geriatric Depression Scale. The Daily Spiritual Experience Scale, the Systems of Belief Inventory (SBI-15R) and a number of standardized tests examining attention were administered: Trail Making Test-Part A (TMT-A) time to completion, the Digit Span (WAIS-III) greatest forward span, the Ruff 2 & 7 Selective Attention Test automatic detection speed (ADS) and controlled search speed (CSS).

**Results:**

indicated that there was a statistically significant difference between the control group and the stroke group in attention. No statistically significant difference was found between the two groups regarding the levels of spirituality and religiousness.

**Conclusions:**

Although spirituality and religiousness may be related with quality of life, cognitive functioning such as attention does not seem to be influenced by these variables one year post-stroke. Future research should further investigate the possible influence of the abovementioned factors in post-stroke recovery and rehabilitation.

